# Life in biophotovoltaics systems

**DOI:** 10.3389/fpls.2023.1151131

**Published:** 2023-08-08

**Authors:** Shangjie Ge-Zhang, Taoyang Cai, Mingbo Song

**Affiliations:** ^1^ College of Science, Northeast Forestry University, Harbin, China; ^2^ Aulin College, Northeast Forestry University, Harbin, China; ^3^ College of Forestry, Northeast Forestry University, Harbin, China

**Keywords:** BPV, biological materials, algae, separate products, review

## Abstract

As the most suitable potential clean energy power generation technology, biophotovoltaics (BPV) not only inherits the advantages of traditional photovoltaics, such as safety, reliability and no noise, but also solves the disadvantages of high pollution and high energy consumption in the manufacturing process, providing new functions of self-repair and natural degradation. The basic idea of BPV is to collect light energy and generate electric energy by using photosynthetic autotrophs or their parts, and the core is how these biological materials can quickly and low-loss transfer electrons to the anode through mediators after absorbing light energy and generating electrons. In this mini-review, we summarized the biological materials widely used in BPV at present, mainly cyanobacteria, green algae, biological combinations (using multiple microorganisms in the same BPV system) and isolated products (purified thylakoids, chloroplasts, photosystem I, photosystem II), introduced how researchers overcome the shortcomings of low photocurrent output of BPV, pointed out the limitations that affected the development of BPV’ biological materials, and put forward reasonable assumptions accordingly.

## 1 Introduction

With the progress of technology and the expansion of population, the demand for energy is increasing (Martiskainen, 2007; Geels et al., 2018; Rahman, 2020; Berrill et al., 2021; Khan et al., 2021). However, the traditional way of obtaining energy, especially thermal power generation, consumes a large amount of fossil fuels with low conversion efficiency, emits greenhouse gases and harmful gases, and produces a lot of pollution (Penghao et al., 2019), which is undoubtedly contrary to the contemporary green development (Hayat et al., 2019; Lin and Xu, 2020). On the other hand, the limited traditional technology can’t meet the huge energy demand (Kalair et al., 2021). As a new energy acquisition system, photovoltaic power generation can directly convert solar radiation energy into electric energy without mechanical wear (Zhang et al., 2020; Ang et al., 2022). Because photovoltaic panels are easy to assemble, can supply energy on demand (Liu et al., 2019) and are environmentally friendly (Narasipuram et al., 2018; Abid et al., 2019; Firat, 2019), it is a potential mainstream green renewable energy technology, which has attracted more and more researchers’ attention.

As the energy source of photovoltaic power generation, solar energy is widely distributed and almost unlimited. However, the distribution of solar energy is uneven. Therefore, how to capture and utilize solar energy efficiently and apply photovoltaic power generation to places with insufficient direct sunlight is the direction that international scholars have been striving for (Qinghui and Jun, 2009; Cotfas and Cotfas, 2019; Venkateswari and Sreejith, 2019; Al-Shahri et al., 2021). At present, the common methods include installing solar automatic tracking device (Sidek et al., 2017; Mamodiya and Tiwari, 2021; Wu et al., 2022), optimizing photovoltaic energy storage technology (Ibrahim et al., 2021; Symeonidou et al., 2021; Fagiolari et al., 2022; Jiang, 2022), changing the material composition of solar panels (Rong et al., 2018; Lin et al., 2020), introducing concentrators (Indrasari and Fahdiran, 2020; Obaid et al., 2021), and introducing photosynthesis mechanism (Jawre and Center, 2018; Yadav and Nair, 2021). Among them, as a relatively new field, the research frequency of BPV technology has increased significantly in recent years. BPV is a new technology that applies natural photosynthesis to solar power generation, that is, photosynthetic autotrophs or their parts are used to collect light energy and generate electricity (Tian et al., 2021). Compared with silicon-based solar panels, bio-based solar panels are easier to capture light and produce less pollution in the manufacturing process. Similar to microbial fuel cells, BPV has the advantages of self-assembly, self-repair and natural degradation because photosynthetic organisms can reproduce themselves (Kornienko et al., 2018; Milano et al., 2019). Compared with microbial fuel cells, BPV doesn’t need to continuously provide organic compounds as substrates to start and operate (Rosenbaum et al., 2010; Xiao and He, 2014).

**Figure 1:**
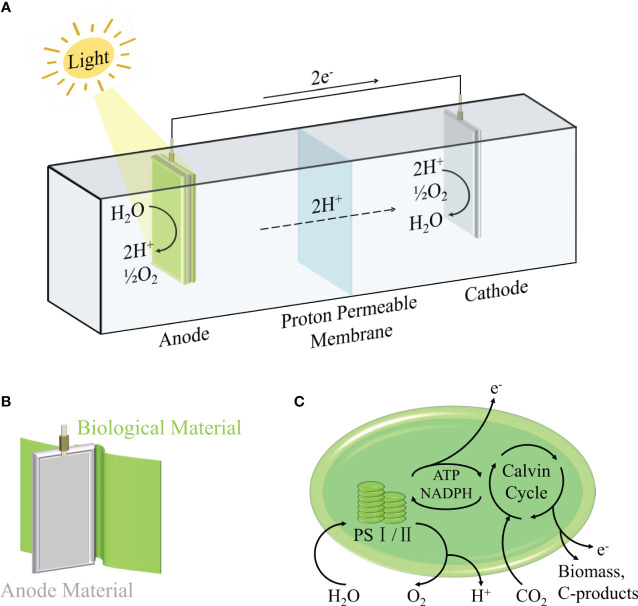
Schematic representation of a biophotovoltaic system **(A)**, Schematic diagram of anode coated with biological materials **(B)** and electron generation model of biological photosynthesis **(C)**.

**Figure 2:**
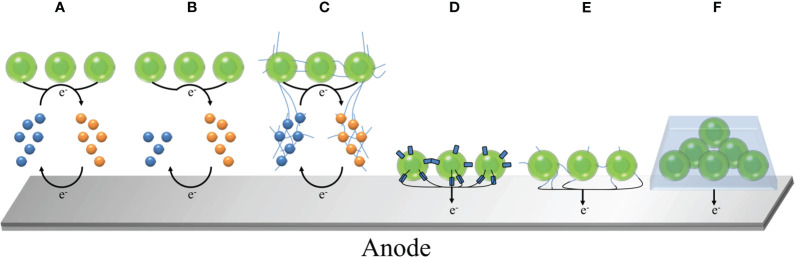
Mechanism of microbial electron transfer in BPV. **(A)** IEET involving recyclable electronic media; **(B)** IEET involving non-recyclable electronic media, such as H_2_; **(C)** IEET of the interaction between electronic media and conductive nanowires (nanonets); **(D)** DEET mediated by biological membrane proteins; **(E)** DEET mediated by conductive nanowires (nanonets)/fimbriae; **(F)** Extracellular polymer-mediated DEET.

Cyclic voltammetry (measuring the current-voltage curve at the fixed electrode by symmetrical triangle potential scanning), chronoamperometry (measuring the current-time curve at a constant voltage) and power curve are commonly used to evaluate the power output of the system (Tschörtner et al., 2019). This is usually regulated by many factors (Wey et al., 2019), including two-electrode or three-electrode system (Saper et al., 2018; Chouler et al., 2019), the choice of electrode materials and morphology (Bombelli et al., 2012; Anam et al., 2021; Wang et al., 2021), electron transfer path (Bradley et al., 2012; Cereda et al., 2014; Park and Song, 2021), ambient temperature (Ciniciato, 2022) and ambient light intensity (Thong et al., 2019; Morlock et al., 2021; Torabi et al., 2021). Among them, the selection of photosynthetic organisms is a major factor affecting the efficiency of BPV. In the general BPV system, the electrons generated by photosynthesis are transferred to the anode, and finally transferred to the cathode through wires, forming a loop current. As shown in **Figure 1**, the anode is used to drive water oxidation by light energy to generate electrons and hydrogen ions, which requires the completion of biological materials, including prokaryotes (mainly cyanobacteria), eukaryotes (green algae, diatoms, etc.), biological combinations (algae and other bacteria), isolated products (purified thylakoids, chloroplasts, photosystem I, photosystem II), which will be described in detail in the second section. Part of the generated electrons will be transferred to the extracellular environment to reduce the anode. For the cathode, the higher electrode potential will drive the current through the external circuit, so that the diffused hydrogen ions and oxygen get electrons to generate water again, or reduce to hydrogen (Saper et al., 2018). It is worth noting that there are various strategies for the transfer of electrons from biological materials to anodes in BPV, which are mainly divided into two categories (Bühler et al., 2021; Syed et al., 2021; Thapa et al., 2021): indirect extracellular electron transfer (IEET) through electron mediator and direct extracellular electron transfer (DEET) through various conductive current-carrying substances (**Figure 2**). Compared with IEET, DEET is faster and not limited by diffusion rate, but requires close and effective contact between organism and electrode (McCormick et al., 2015; Thirumurthy et al., 2020).

In this mini-review, the principle and advantages of BPV are reviewed, and the evaluation methods and related parameters of BPV efficiency are listed. In particular, this paper focuses on how different biological materials and their separated products enhance and effectively guarantee BPV power generation technology (Section 2). At the end of the review, the future direction of biological materials reinforced photovoltaic system technology is prospected.

## 2 Biological materials used in BPV

### 2.1 Cyanobacteria

At present, cyanobacteria is the most commonly used material in the research of BPV. As a single-celled prokaryote, cyanobacteria have relatively simple cell membrane arrangement, which is conducive to electron output, and contains chlorophyll a, lutein, carotene, phycobilirubicin and other pigments, which has a good light adaptation mechanism (Komárek, 2018; Hirose et al., 2019). In addition, its nutritional requirements are simple, and only light, water, carbon dioxide and inorganic salts are needed to realize photoautotrophy (Zheng et al., 2022). Among cyanobacteria, *Synechocystis* sp. PCC6803 is the most commonly used for BPV research, which may be due to the early completion of the whole genome sequencing, and it is an important model widely used for photosynthesis and cyanobacteria biology research at present (Kaneko and Tabata, 1997; Liu and Pakrasi, 2018; Mills et al., 2020; Schneider et al., 2022). In addition to autotrophic growth, *Synechocystis* sp. can also use glucose for heterotrophic growth, that is, light activated heterotrophic growth (LAHG) (Huokko et al., 2019; Solymosi et al., 2020), in the absence of light, which ensures the service life and self-healing characteristics of BPV, making it reliable and stable to use BPV for long-term power generation. According to the research results of Hasan et al. (2017a), the photocurrent density produced by cyanobacteria is higher than that of eukaryotic algae. This may be due to different positions of photosynthesis in cells, different arrangement of cell inner membrane and different difficulty of electron transfer. That is to say, the photosynthetic apparatus of eukaryotic algae is located in the chloroplast, and the chloroplast membrane blocks the transfer of electronic media to some extent. All the above reasons explain why most researchers are keen to choose cyanobacteria as the main research object of BPV.

Researchers have been working on the metabolism and genetic manipulation of cyanobacteria for a long time (Clerico et al., 2007). At present, using natural mutation or genetic engineering of cyanobacteria to achieve better electron transfer or light absorption is one of the hot research directions (Hitchcock et al., 2020; Sinha, 2021). *Synechocystis* sp. PCC6803, as an excellent genetic tool, can construct a mature scheme of mutants with multiple chromosome deletions and insertions, as well as more precise operations, such as introducing single point mutations into specific genes (Lea-Smith et al., 2014). Kusama et al. (2022) used *Synechocystis* sp. PCC6803 mutant, slr0688i, with outer membrane deletion, found that outer membrane deletion enhanced EET effect, and compared with wild strains, its photocurrent increased by an order of magnitude. Sekar et al. (2016) genetically modified *Synechococcus elongatus* PCC 7942 to express the unnatural redox protein outer membrane cytochrome S (OmcS), which improved the EET ability and increased the photocurrent by 9 times compared with the wild type. In addition, the ability to convert carbon dioxide, NADPH and ATP into products during dark reaction can be regulated by codon optimization, so as to minimize the loss of free energy in large-scale photobioreactor (Angermayr and Hellingwerf, 2013).

Some researchers also focus on the influence of environment on the efficiency of algae photocurrent generation, including external environment and culture environment. As NADPH is one of the mediators of EET effect of cyanobacteria, it is undoubtedly feasible to add exogenous NADP^+^ to cyanobacteria (Sandmann and Malkin, 1983; Shlosberg et al., 2021a; Yang et al., 2022). Park et al. (2019) developed a simpler external environment-assisted electron transfer method, which directly added carbon nanotubes (CNTs) to cyanobacteria suspension containing 2,5-dimethyl-1,4-benzoquinone mediator. With this method, electrons can be quickly transferred to the anode along the CNTs network. However, due to the extremely strong absorbance of CNTs, excessive addition of CNTs will lead to a sharp drop in photosynthetic efficiency, so it is necessary to control the trace amount of CNTs. By applying slight pressure to *Synechococcus elongatus* PCC7942, Okedi et al. (2020) think that increasing the surface area of cyanobacteria can increase the EET rate, and for rod-shaped cells, the increase of cell area caused by cell elongation is enough to compensate for the decrease of mass transfer coefficient. In addition, the mediators used by researchers include 1,4-benzoquinone (Lee et al., 2020), 2,6-dichlorobenzoquinone (Longatte et al., 2016; Longatte et al., 2017), p-phenylbenzoquinone (Longatte et al., 2015), p-benzoquinone (Cevik et al., 2021), etc. In terms of growth and culture environment, *Synechococcus elongatus* PCC7942 cultured by Gonzalez-Aravena et al. (2018) under the condition of iron limitation showed stronger discharge activity and stronger mediator-free interaction between cyanobacteria and electrodes compared with the condition of sufficient iron. In addition, iron-limited treatment can significantly reduce ferricyanide in the dark, which is 6 times of that under the condition of sufficient iron culture. Liu et al (Liu and Choi, 2021). used intracellular *in-situ* biosynthesis technology. By culturing *Synechocystis* sp. PCC 6803 in BG-11 medium with a small amount of chloroauric acid (HAuCl_4_) solution for 16 h, the random distribution of Au-NPs on the cell membrane was completed. The appearance of Au-NPs promoted the generation of photoinduced electrons and EET at the cell electrode interface. The maximum power density of BPV reached 18.8 μW cm^-2^, which was 33.6 times that of the case without nanoparticles. After about 120 h of culture, only < 8% of cyanobacteria died, which indicated the feasibility and stability of environmental modification of biological materials in BPV. In addition, *Synechocystis* sp. PCC6803 strain can also be directly grown on a transparent and conductive anode (indium tin oxide coated polyethylene terephthalate), the maximum 10.3 mW m^-2^ total power output recorded under 10 W m^-2^ white light can be obtained, and the need for external media can be avoided (McCormick et al., 2011). The environmental changes of cyanobacteria in the above experiments have no obvious influence on the service life and stability of BPV.

### 2.2 Eukaryotic algae

Eukaryotic algae have also been studied, among which green algae is the most typical example. In the aspect of mediator environment, Anderson et al. (2016) tested the single-celled green algae *Chlamydomonas reinhardtii* strains *cw15* (cc-1883), *cw92* (CC-503), *sta6rbo1* (CC-4348), *sta6rbo1* (STA6)-c2 (CC-4565) and *sta6rbo1* (STA6)-c4 (CC-4566) respectively, showed that plasma membrane NADPH oxidase is an important component of *Chlamydomonas reinhardtii* light-dependent power generation, and the expression of NADPH oxidase can help electrons to cross the plasma membrane and form superoxide anion from oxygen. Shlosberg et al. (2021b) also studied the electron transfer mechanism of *Dunalliela salina*, thought that NADPH was its main endogenous medium, and there was a synergistic effect with FeCN. The synergy of two mediators could significantly enhance the photocurrent (beyond the sum of the photocurrent enhancement values of adding media separately). Similar to the research of Liu et al (Liu and Choi, 2021). above, Kuruvinashetti et al. (2022) successfully internalized Au-NPs into *Chlamydomonas reinhardtii* (CC-125Wild-type MT+[137C]) by dropping chloroauric acid (HAuCl_4_) at the middle logarithmic stage. On the one hand, it increased the light absorption in a wider visible spectrum range, on the other hand, it was more efficient. *In-situ* biosynthesis of Au-NPs to most algae is feasible, because nano-sized Au particles have good biocompatibility (Chellapandian et al., 2019; Kang et al., 2020; Nejati et al., 2022). In addition, the fluo-electrochemical setup designed by Beauzamy et al. (2020) can monitor the behaviors of photosynthetic chains and redox mediators when they are exposed to each other, creating technical conditions for finding redox mediators with less toxicity and quenching characteristics in the future. Other studies think that the study of photoprotection mechanism should be the focus of improving photosynthetic productivity of algae in the future, including alternative electron transport (AET) and nonphotochemical quenching (NPQ) (Andersson et al., 2019; Nawrocki et al., 2019; Ware et al., 2020).

There are relatively many researches on cyanobacteria and green algae in BPV, but there are also a few researches on other algaes. Laohavisit et al. (2015) have studied the electron transfer of *Phaeodactylum trichonutum* (strain 1055/1) and *Thalassiosira pseudonana* (strain 1085/12), and found that the high expression of NADPH oxidase is also the key to make marine diatoms produce extracellular superoxide anion and increase BPV electron outflow. In addition, the superoxide anion and output power can also be adjusted by changing the lighting conditions. Through the in-depth exploration of marine diatom *Thalassiosira oceanica* CCMP1005, Diaz et al. (2019) thought that extracellular superoxide is a by-product in the process of electron transfer, and the oxidation of NADPH completes the redox dynamic balance of cells. Shlosberg et al. (2022) studied six complete macroalgae individuals (green alga: *Ulva*, *Cladophora*; red alga: *Gracilaria*, *Jania*; Brown alga: *Padina*, *Stypopodium*) has made a comprehensive study, and it is found that macroalgae can generate a current of >50 mA cm^-2^ at most, and NADPH, Fe(CN)_6_ and hydroxyl particles may be potential regulatory media for electron transfer.

### 2.3 Biological combination

Considering that BPV composed of pure photosynthetic autotrophic microorganisms has high photosynthetic efficiency, compared with microbial fuel cells, its electricity-generating activity is weak and its electricity collection efficiency is low (Elshobary et al., 2021). Therefore, BPV can be combined with microbial fuel cell to enhance the output power. It is generally composed of an autotrophic microorganism and a heterotrophic microorganism: autotrophic microorganisms produce organic substrates through photosynthesis, while heterotrophic microorganisms consume substrates through respiration to produce photoresponse electric energy (Laureanti and Jones, 2016; Pankratova et al., 2022). Because of the combination of microbial fuel cells, the biological combination BPV can also input foreign organic substrates, which makes the BPV still be able to continuously output current when there is no light (Sun et al., 2019).

The most selected biological combinations are cyanobacteria (generally *Synechocystis* sp. PCC6803, autotrophic organism) and *Shewanella oneidensis* (heterotrophs) (Tsujimura et al., 2001; Liu and Choi, 2017; Mekuto et al., 2020). Liu et al. (2018) closely attached the cyanobacteria species most commonly used in BPV research to Shewanella. By inoculating *Synechocystis* sp. PCC6803 on *Shewanella oneidensis* MR-1 biofilm, a BPV with a current of 8 μA cm^-2^ for up to 13 days was prepared without the input of exogenous organic substances. In addition, they also improved the chamber structure and improved the adhesion of microorganisms. Liu et al (Zhu et al., 2019). used additive manufacturing technology, by 3D printing a hydrogel bio-layer containing *Synechocystis* sp. PCC6803 on *Shewanella oneidensis* MR-1, the power of about 13 μW cm^-2^ was obtained. Zhu et al (Mohammadifar et al., 2020). developed a double-strain BPV with an average power density of 135 mW m^-2^, which can run stably for more than 40 days in continuous fed-batch culture. Two bacteria in BPV are *Synechococcus elongatus* UTEX 2973 and *Shewanella oneidensis* MR-1. The d- lactic acid is used as the carrier to complete the energy conversion between microorganisms. Cyanobacteria absorb light energy to synthesize energy carrier d- lactic acid, while Shewanella oxidizes d- lactic acid to generate electricity, thus completing the process of energy conversion from light energy to chemical energy and then to electric energy.

In the field of system structure of BPV, *Shewanella oneidensis* MR-1 and *Synechocystis* sp. PCC6803 are also used, while Mohammadifar et al (Sun et al., 2020). designed a BPV using solid-phase equipment components, with solid anolyte, catholyte and salt bridge. In the anode, agar is used to separate two microbial communities, allowing only the exchange of nutrients and gases, thus reducing the competition among microorganisms. This kind of solid-phase equipment reduces the environmental occupancy rate and improves the feasibility of BPV in practical application. Sun et al (Liu and Choi, 2019). made a three-electrode BPV with *Chlorella vulgaris* and *Rhodopseudomonas palustris* as biological materials, and found that the potential required to extract electrons from the latter was lower, and had lower dependence on external electron mediator. Besides biological combination, BPV can also be combined with other electrical devices to improve current output efficiency, such as self-charging supercapacitors (Das et al., 2018; Liu and Choi, 2020a; Liu and Choi, 2020b; Powell et al., 2021; Alajmi et al., 2022; Pankratova et al., 2022) or more complex boost converters (Hill and Bendall, 1960; Singh and Pandit, 2013; Oseyemi et al., 2022).

### 2.4 Separated products

In most cyanobacteria, the main light-dependent electrification reaction takes place in photosystem (PS), which is the functional unit responsible for light absorption distributed on the thylakoid membrane (Perez-Boerema et al., 2020). It is a compound composed of chlorophyll, carotenoids, lipids and protein. The particles of photosystem I (PS I) (Qin et al., 2019; Sheng et al., 2019; Naschberger et al., 2022) are small and mainly distributed in the non-stacked part of thylakoid membrane. Photosystem II (PS II) (Pinhassi et al., 2016; Kosourov et al., 2020) is an enzyme that catalyzes light-induced water oxidation, with large particles, mainly distributed in the stacking part of thylakoid membrane. Other subcellular parts isolated and purified by photosynthetic organisms, such as chloroplasts, can also be used for BPV, and *Spinacia oleracea* (Magnuson, 2019; Pankratov et al., 2020) is widely used in experiments. This is due to the high chlorophyll content in the leaves of *Spinacia oleracea*, which is easy to obtain and grind. It should be noted that BPV based on isolated products has a relatively short lifetime due to the lack of other cell components necessary for cell repair.

The change of thylakoid membrane caused by gene mutation is one of the research focuses (Lea-Smith et al., 2013; Larom et al., 2015; Yildiz et al., 2019; Wey et al., 2021). The research of Larom et al (Viola et al., 2021). showed that the mutation of Photosystem II D1-K238E in *Synechocystis* sp. PCC6803 was sensitive to the increase of photocurrent (other mutations of K238 or other residues in the same vicinity were not significant in the decrease of cytochrome C). Viola et al (Hartmann et al., 2020). used kanamycin resistance cassette to partially replace endogenous petE gene to obtain *Synechocystis* ΔPC mutant, and a series of comparative experiments showed that the distribution of electron transfer between respiration and photosynthesis did not depend on the existence of one of the two intracavity electron carriers (plastocyanin, cytochrome c_6_) alone. In addition, Hartmann et al (Pankratov et al., 2020). modified the purified PS II by externally adding phycobilisome, which broadened the total absorbance in the visible range. We think it is also possible to edit related genomes to achieve the same effect.

Besides gene level, reasonable BPV’ design (including electrode selection, mediator types, etc.) is also the key to improve photocurrent (Lea-Smith et al., 2016; Antonacci and Scognamiglio, 2019; Morlock et al., 2021). Morlock et al (Pankratova et al., 2018). firstly extracted and purified PS I from *Thermosynechococcus elongatus* to obtain PS I trimer, and then the three-dimensional rGO electrode structure is constructed by spin coating technology, which has the advantage that the electrode thickness can be controlled by controlling the number of spin coating. Pankratova et al (Calkins et al., 2013). fixed thylakoid films on graphene surface by electro-reduction, electro-deposition and amino aryl functionalization, and obtained a photocurrent density of 5.24 ± 0.50 μA cm^–2^ without mediator, which has the advantages of simple design, sustainability and low cost. Carbon nanotubes with similar properties to GO are also common suitable materials (Takeuchi et al., 2018; Adachi et al., 2019; Pankratov et al., 2019; Yun et al., 2022). Adachi et al (Bunea et al., 2018). designed a BPV composed of thylakoid membrane (anode biological material) -[Ru(NH_3_)_6_]^3+/2+^ (mediator) -bilirubin oxidase (cathode), which showed an open circuit voltage of 0.61 V and a maximum power density of 50 μw cm^-2^. Bunea et al (Cevik et al., 2018). also compared the photocurrent generated by the anode BPV of thylakoid membrane with [Ru(NH_3_)_6_]^3+^ or [Os(2,2’-bipyridine)_2_-poly(*N*-vinyl imidazole)_10_Cl]^+/2+^ as mediator, and also confirmed [Ru(NH_3_)_6_]^3+^. Cevik et al (Hasan et al., 2017b). comprehensively considered the choice of mediator and electrode. Cytochrome C was first crosslinked to the gold electrode coated with P(DTP-Ph-NH_2_) conductive polymer, then the thylakoid membrane was attached, and bilirubin oxidase was immobilized on the cathode. At the current density of 15 mA m^-2^, the maximum power generation is 4.9 mW m^-2^.

As another commonly used anode biological material, chloroplast can be easily separated and fixed on the electrode, and can independently perform energy conversion and electron transfer under light (Grattieri et al., 2020; Howe and Bombelli, 2020; Okedi and Fisher, 2021; Chin et al., 2022). Because it is simple and economical to separate and purify chloroplasts, the method of changing biological materials regularly can make up for the disadvantage of BPV caused by the inability of chloroplasts to reproduce themselves. However, the low electron transfer efficiency of chloroplasts has become an urgent problem to be solved. Grattieri et al (Weliwatte et al., 2021). used ethylene glycol diglycidyl ether (EGDGE) as a cross-linking agent to deposit chloroplasts on anode in one step to enhance the collection of photoinduced electrons, and obtained 5 times of biological photocurrent. In Weliwatte et al.’ s experiment (Lv et al., 2022), poly (dihydroxyaniline) (PDHA), as the external redox mediator and the fixed matrix of the interface, was mixed with ethylene glycol diglycidyl ether (EGDGE) and chloroplast to prepare the anode, which increased the photocurrent density by 2.4 times. When the mixture of PDHA and EGDGE was first dripped, and then the chloroplast was dripped after drying, the increment reached 4.2 times. In addition, similar to the above, anode materials with excellent conductivity are also relatively mature research fields (Poddar et al., 2020; Shiyani and Bagchi, 2020; Christwardana et al., 2021). By studying the electron transfer ability of chlorophyll in *Chlorella vulgaris*, Christwardana et al. (158) designed CNTs/chlorophyll anode by coating technology, and achieved 6 times photocurrent.

## 3 Summary and outlook

BPV is a new technology developed on the basis of photovoltaic industry. It not only inherits the advantages of traditional PV, such as using solar energy resources, not emitting harmful gases and having no noise, but also solves the disadvantages of high pollution and high energy consumption in the manufacturing process of crystalline silicon battery. It can be said that it is the most ideal renewable energy power generation technology for sustainable development. In this mini-review, we focus on how different biological materials and their isolated products enhance and effectively guarantee BPV technology. The development of BPV is not perfect, and there are some shortcomings, such as low conversion efficiency, great influence of climate, and its related theories are still immature. Therefore, it is of great significance to deeply study the related issues of BPV. In view of some hot issues, we put forward the following prospects:

(1) Actually, the mechanism of the interaction between biological materials and the external environment and biological materials in BPV is not clear, and the physiological theory still needs to be explored. Moreover, although most methods are similar, due to the lack of uniform standards (such as standard culture environment and standard system setup), most horizontal comparisons are difficult to conduct uniformly and have no reference value.
(2) Compared with traditional solar photovoltaic, the conversion efficiency of BPV is too low. Therefore, it is necessary to use genetic engineering methods to increase the current output of photosynthetic organisms, which includes not only stronger photosynthesis and more mediators, but also changes in cell morphology and structure, but it needs the support of a sound biological mechanism.
(3) More diverse biological combinations should be considered, not only the combination of “autotrophic strains@heterotrophic strains”, but also other combinations including “strains that can secrete mediators@strains with low electron transfer rate due to absence of mediators”, “strains that like strong light photosynthesis@strains that like weak light photosynthesis” and so on.
(4) It is easy to notice that the size and distribution of intracellular nanoparticles are irregular when nanoparticles are introduced by *in-situ* intracellular biosynthesis technology. If the size and position of nanoparticles can be precisely controlled, it is believed that the electron mobility can be greatly improved.
(5) The structural improvement of BPV may be a simpler and more obvious enhancement. As biological materials need to be in close contact with the anode, the three-dimensional anode with more effective surface area is the potential direction. Cheap and sustainable carbon-based electrodes are currently one of the most widely used anodes, but how to maintain high light transmittance is a difficult problem to be solved.

## Author contributions

SG-Z: Writing – original draft, Writing – review and editing, Investigation, Visualization, Methodology. TC: Writing – original draft, Writing – review and editing, Investigation, Visualization, Methodology. MS: Resources; Supervision, Writing – review and editing.
